# Disordered array of Au covered Silicon nanowires for SERS biosensing combined with electrochemical detection

**DOI:** 10.1038/srep25099

**Published:** 2016-04-26

**Authors:** Annalisa Convertino, Valentina Mussi, Luca Maiolo

**Affiliations:** 1 Institute for Microelectronics and Microsystems, C.N.R., via del Fosso del Cavaliere 100, 00133 Rome, Italy; 2 Institute for Complex Systems, C.N.R., via del Fosso del Cavaliere 100, 00133 Rome, Italy

## Abstract

We report on highly disordered array of Au coated silicon nanowires (Au/SiNWs) as surface enhanced Raman scattering (SERS) probe combined with electrochemical detection for biosensing applications. SiNWs, few microns long, were grown by plasma enhanced chemical vapor deposition on common microscope slides and covered by Au evaporated film, 150 nm thick. The capability of the resulting composite structure to act as SERS biosensor was studied via the biotin-avidin interaction: the Raman signal obtained from this structure allowed to follow each surface modification step as well as to detect efficiently avidin molecules over a broad range of concentrations from micromolar down to the nanomolar values. The metallic coverage wrapping SiNWs was exploited also to obtain a dual detection of the same bioanalyte by electrochemical impedance spectroscopy (EIS). Indeed, the SERS signal and impedance modifications induced by the biomolecule perturbations on the metalized surface of the NWs were monitored on the very same three-electrode device with the Au/SiNWs acting as both working electrode and SERS probe.

Surface enhanced Raman scattering (SERS) occurring at the surface of rough noble metals is an analytical technique allowing ultrahigh sensitivity and *in situ* recognition of molecules even in a liquid environment. These features and the strong progress in the development of new lasers and compact user-friendly spectrometers make the SERS method an appealing analytical tool in diverse fields, including medicine, environmental monitoring and trace chemical analysis^1^,^2^,^3^,^4^,^5^,^6^.

Actually, the development of effective SERS active materials is the key aspect for the successful transition of the technique to a practical biological and chemical sensing methodology. The advances in nanofabrication technologies have led to the emergence of a myriad of novel SERS substrates, including colloidal metal (essentially Au or Ag) nanoparticles (NPs)^7^,^8^, assembly of metal nanostructures on different surfaces^9^,^10^,^11^,^12^, metal decorated porous materials^13^,^14^, metal films evaporated on nanosphere-coated substrates^15^. Although these nanomaterials show large Raman enhancement ability, most of them are too technologically demanding and expensive to be used to fabricate large quantities of substrates for practical applications with high reproducibility and long term storage properties.

Silicon nanowires (SiNWs) decorated with metal NPs are now being intensively investigated as SERS substrates due to their high enhancement factors and improved detection limits recently demonstrated^16^,^17^,^18^,^19^,^20^,^21^,^22^. These structures offer the opportunity to combine huge surface-to-volume ratios, facile surface modification, and full compatibility to the well-established silicon technology. It is worth to highlight that successful fabrication of ultrasensitive electrochemical and optical sensors based on SiNWs has been recently demonstrated for biomolecule and gas detection^23^,^24^,^25^,^26^,^27^,^28^. Thus the development of SERS sensors based on SiNWs would widen the potential applications of this sensing platform permitting the use of different transduction signals.

We have recently showed as a highly disordered array of SiNWs covered by a film of Au (Au/SiNWs) can act as performing electrochemical biosensor *via* electrochemical impedance spectroscopy (EIS)^29^. The key benefit is that disordered SiNWs can be easily obtained through high yield and large-area fabrication techniques as well as relative low temperature (350 °C) procedures compatible with polymeric films, i.e. Polyimide, and glasses. The accommodation of SiNW based biosensors on plastic and glass provides an effective and cheap method to integrate these sensing nanoelements into macroscopic and/or commercial readout devices including assay plates and microscope slides implementable in benchtop microscopies. The idea of this work is directed to investigate the very same composite structure as Raman probe, allowing ultimately the combination of SERS detection with EIS control. This combination of an optical and electrochemical signal transduction in the same Au/SiNWs can offer an improved method to identify and quantify the bio-analytes over either technique independently. Indeed, EIS is widely used in diagnostic biosensing, because it allows detecting a wide range of analytes with high sensitivity by using chip and miniaturized devices^30^. On the other hand, the impedance biosensors do not provide chemical identification of the analyte and can reveal also non-target biomolecules stuck to the probe causing a false positive signal. This is a critical issue when biological fluids containing a large background of nonspecific interferents (blood, saliva etc.) are analyzed. The SERS offers the complementary chemical information due to the ability to identify analytes from unique Raman signatures reducing the issue of false positive signals.

To show the potential of this approach SiNWs, few microns long, were grown by plasma enhanced chemical vapor deposition (PECVD) and covered by an evaporated thin Au layer, 150 nm thick. Common microscope glass slides were used as substrates to demonstrate these nanostructures can be built via scalable and low-cost fabrication methodology on supports that can be easily integrated into Raman spectrophotometers. The properties of Au/SiNWs as Raman probe and their sensing performance were studied via the ligand-receptor binding of biotin-avidin system. Finally we fabricated a three-electrode device with the working electrode (WE) consisting of the investigated Au/SiNWs and showed the detection of the biotin-avidin interaction through two different methods of transduction: SERS and electrochemical.

## Results and Discussion

### Material characterization

**Figure 1:**
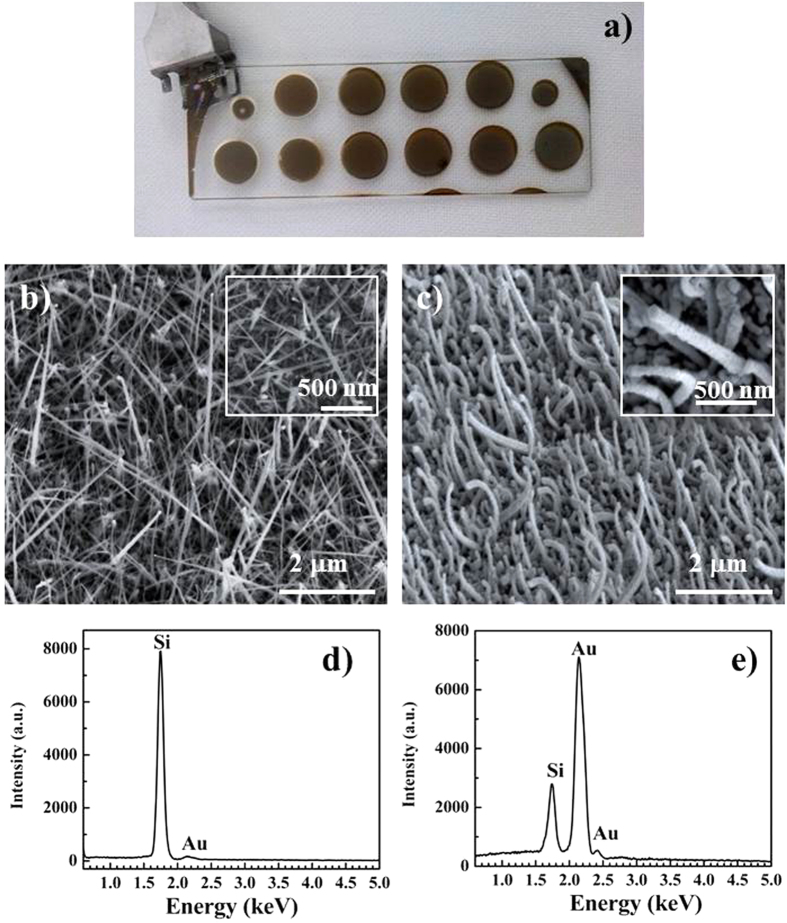
Photograph of the Au/SiNWs grown on microscope slide (**a**). Tilted view (ca. 50°) SEM images of the as grown SiNWs (**b**) and after Au coverage (**c**). EDX spectra of the as grown SiNWs (**d**) and after Au coverage (**e**).

A representative photograph of the Au/SiNWs grown on a microscope slide is in Fig. 1a. The brown circles are the large active areas formed by NWs. The materials are characterized by scanning electron microscopy (SEM) and energy-dispersive X-ray (EDX) analysis. The SEM images of these structures as grown and after Au coverage are in Fig. 1b,c, respectively. These images reveal a dense ensemble of disordered and randomly oriented NWs. The as grown SiNWs (see Fig. 1b) are long around 2–3 μm with an average diameter at the bottom of about 40–70 nm and show a tapered shape. The size of the NWs was determined by combined measurements from plan and cross views. The EDX spectrum of the pristine NWs in Fig. 1d shows the strong Si peak related to the Si NWs and a weak Au peak due to the presence of the catalyst particles that are visible as bright spots at the tip of the longer NWs in Fig. 1c. After evaporating the Au layer, in Fig. 1d we observe Au/SiNWs with a more cylindrical shape and an increased average radial size, which is around 100–120 nm at the bottom. The Au catalysts particles are no more visible because completely covered by the evaporated metal film and any relevant variation in NW length is evident. In the inset of Fig. 1d, high-resolution image reveals very rough sidewalls, which could favor Raman signal enhancement^16^. The SEM images of the covered NWs (Fig. 1d) show, to a visual inspection, a quite uniform Au coating and the EDX analysis (Fig. 1e) highlights strong Si and Au peaks, corresponding to the Si NW array and the overlaying Au film, respectively.

Since the evaporation is not a conformal coating technique, it is reasonable to expect not completely coated NWs due to some shadowing effects during the metal evaporation. Nevertheless, the Au coverage will result enough to make the surface of the wire ensemble ready to be modified with NHS-biotin and then to interact with avidin, as it will be clear later.

From our morphological study a key feature for biosensing applications can be finally evidenced: the Au/SiNWs form a three-dimensional (3D) macroporous framework and offer a huge surface area to immobilize ligand molecules, easily accessible to vapors and liquids containing biomolecule analyte.

### Effects of the NW morphology on the optical properties

Before performing Raman experiments, we investigated the optical properties of the Au/SiNW composite system. The effects of morphology on the optical properties of semiconductor NW arrays are well known: the light passing through a NW forest undergoes multiple scattering events from each NW. The scattering events fold the light path many times in a random walk inside the NW forest causing an enhancement of the light absorption with consequent drastic reduction of the reflectivity at the frequencies absorbed by the NW material, i.e. light trapping^31^,^32^,^33^.

**Figure 2:**
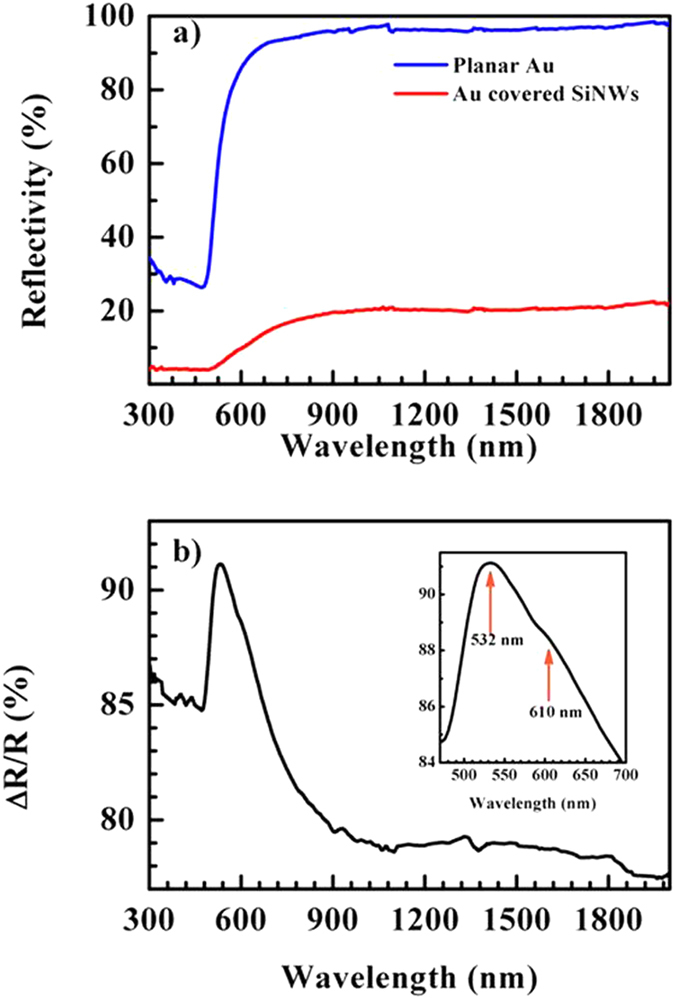
Total integrated reflectivity spectra of the Au/SiNWs (red line) compared with Au planar layer (blue line) (**a**). Relative variation of the reflectivity, 
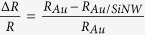
, where *R*_*Au*_ and *R*_*Au/SiNW*_ are the reflectivity of the planar Au and the Au/SiNWs, respectively (**b**). In the inset the enlarged 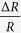 at smaller wavelengths.

To understand the effect of the NW light trapping to the overlying Au film in our composite system, we measured its total integrated reflectivity, *R*, in the spectral range between 200–2000 nm and compared it with that of a reference sample consisting of a planar Au film with the same nominal thickness of the NW coverage. In Fig. 2a, spectra obtained from the Au/SiNWs (red line) and reference sample (blue line) are plotted and compared. The reference sample shows the typical Au bulk reflectance^34^ of around 95% in the infra-red (IR) range with a steep decrease below λ = 650 nm.

Although the profile of *R* for the Au on NWs remains quite similar to that of the planar Au, the intensity of *R* is drastically diminished in the nanostructered sample over the whole spectral range. *R* is in fact reduced down to 20% in the IR and few percent in the visible range. The NW sizes and their broad size distribution, as evidenced by the SEM images, assure indeed a large light scattering at all wavelengths in the IR-visible range by allowing the Au on the NW to absorb significantly more photons than an equivalent volume of bulk material^31^,^32^,^33^. Hence the reduction of the *R* intensity, defined as 
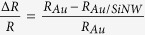
 where *R*_*Au*_ and *R*_*Au/SiNW*_ are respectively *R* of the planar and nanostructured Au, corresponds to the amount of light trapped inside the NW forest and then absorbed by the overlying Au film. If we plot 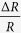
as a function of the wavelength (Fig. 2b) we can observe a quite constant value of about 80% over the whole IR range and a peak of 92% at 532 nm with a shoulder at around 610 nm, inset of Fig. 2b. The shape of the curve and peak position in Fig. 2b suggest the mechanism by which such an absorption occurs could be transverse and longitudinal modes of a plasmonic resonance^35^ probably related to the smaller NWs or NW section, which after Au coverage form Au nanopillars. Anyway, a full understanding of the origin of the plasmonic resonances observed in our composite NWs requires deeper investigations that are beyond the scope of this work and will be performed in more details elsewhere.

### Avidin detection by Raman spectroscopy

To test the capability of Au/SiNWs to act as SERS biosensor we progressively modified their surface by adding cysteamine to achieve a self-assembled monolayer (SAM) coating, NHS-biotin to build the sensing layer, finally avidin as the detected material.

**Figure 3:**
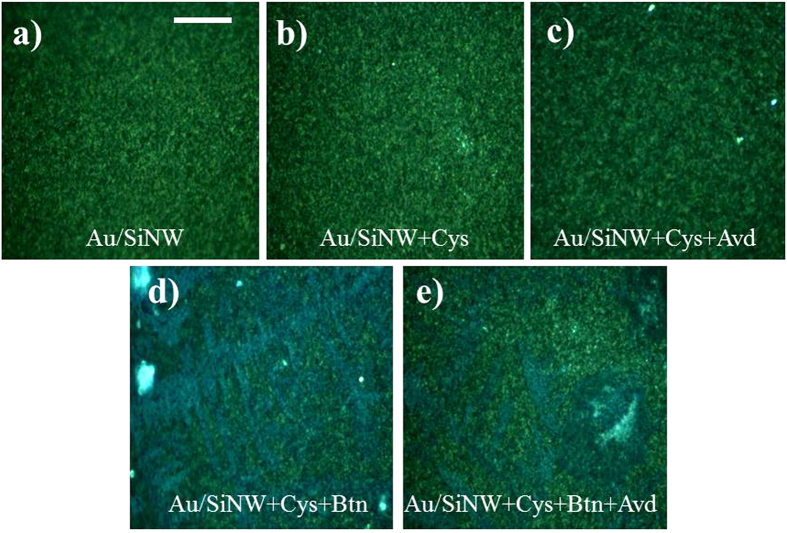
Optical images obtained on the sample at different stages of the functionalization process: the pristine Au/SiNWs (**a**); treated with cysteamine (Au/SiNWs+Cys) (**b**); after NHS-biotin binding (Au/SiNWs+Cys+Btn) (**d**); finally after the immersion in solution with 1 μM content of avidin (Au/SiNWs+Cys+Btn+Avd) (**e**). The image obtained after control experiment: Au/SiNWs modified with sole cysteamine and after exposure to 1 μM content of avidin solution (Au/SiNW+Cyst+Avd) (**c**). Scale bar is 100 μm.

Visual inspection of the sample after each functionalization step shows a change of the surface color as it results in Fig. 3 where optical images of the pristine Au/SiNWs (Fig. 3a), modified with cysteamine (Fig. 3b) and NHS-biotin (Fig. 3d), and after immersing in an avidin solution with a protein content of 1 μM (Fig. 3e) are shown. As control experiment we immersed in the 1 μM avidin solution the Au/SiNWs coated by sole SAM layer, and Fig. 3c shows the related optical image. Any change in the surface aspect is visible after cysteamine SAM formation (Fig. 3b), maybe due to the very small size of the cysteamine monolayer which does not produce any interference effects visible with an optical microscope.

**Figure 4:**
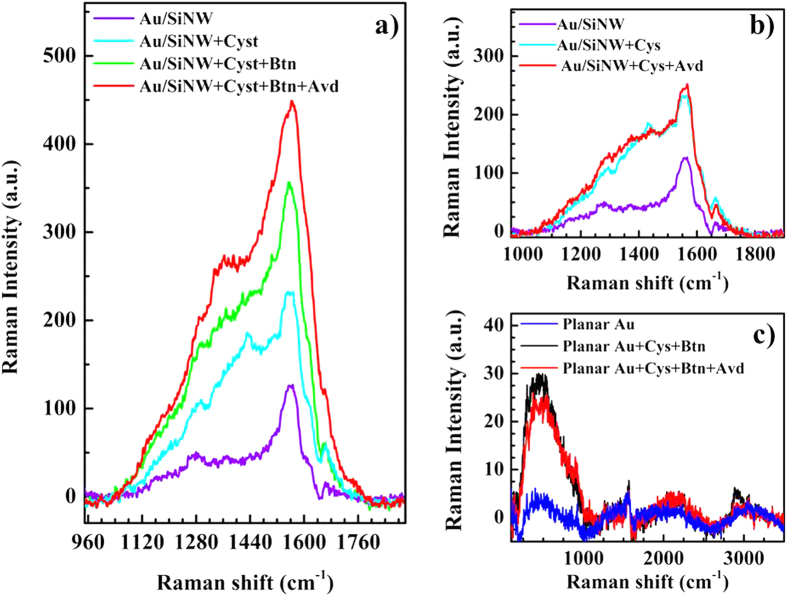
Raman spectra obtained at different stages of the functionalization process of the samples: the pristine Au/SiNW substrate (violet line), after cysteamine modification (cyan line), functionalized with NHS-biotin (green line) and finally treated with avidin at 1 μM content (red line) (**a**). Control tests performed by skipping the treatment with NHS-biotin and immersing only in avidin at 1 μM content (**b**) and on the reference Au planar film (blue line) functionalized with cysteamine + NHs-biotin (black line) and avidin at 1 μM content (red line) (**c**).

After NHS-biotin treatment a change of the surface color from green to blue can be noticed (Fig. 3d), indicating a quite uniform adsorption of the NHS-biotin molecules onto the Au/SiNW surface. Then the blue color becomes attenuated in a very large areas of the samples after avidin exposure (Fig. 3e), whereas any color change can be observed when avidin is added on the sample modified with sole cysteamine (control experiment in Fig. 3c). These observations suggest the avidin immobilization onto the surface of the Au/SiNWs through its capture from the biotin ligand. This is best confirmed by the Raman analysis in Fig. 4a where the Raman spectra corresponding to each step involved in the biosensing experiment are plotted.

We observe a progressive increase of the signal in the spectral range comprised between 1280 and 1450 cm^−1^. In particular, a quite large band at about 1380 cm^−1^ appears in the spectra obtained after the last passage with avidin. This contribution is ascribable to the specific interaction of avidin with biotin^36^ thus demonstrating the stable immobilization of the target molecule onto the treated substrate. In addition, two control experiments were carried out. As first, to confirm that the observed Raman spectra changes in Fig. 4a are due to the specific binding of avidin to the biotin ligand we immersed in the 1 μM avidin solution the Au/SiNWs coated by the sole SAM layer, i.e. without the NHS-biotin ligand. As we can see in Fig. 4b by comparing the Raman spectra of the Au/SiNW coated with cysteamine before (Au/SiNW+Cyst) and after immersion in avidin solution (Au/SiNW+Cyst+Avd), the avidin did not produce any significant change in the signal suggesting a little non-specific binding of the protein to the bare cysteamine modified Au/SiNWs. The second control experiment was realized to evidence the essential role played by the SiNWs in the enhancement of Raman signal. For this purpose the sensing capability to detect avidin was also tested on planar Au film (Fig. 4c), which was coated with cysteamine+NHS-biotin and successively immersed in the 1 μM avidin solution. Any relevant signal variation can be observed between the pristine NWs and after each modification step, suggesting that the nanostructuring of the Au improves the Raman signal. The enhancement of the Raman signal in the Au/SiNWs is most likely due to the occurring of plasmonic resonances observed in the spectrophotometric investigation.

**Figure 5:**
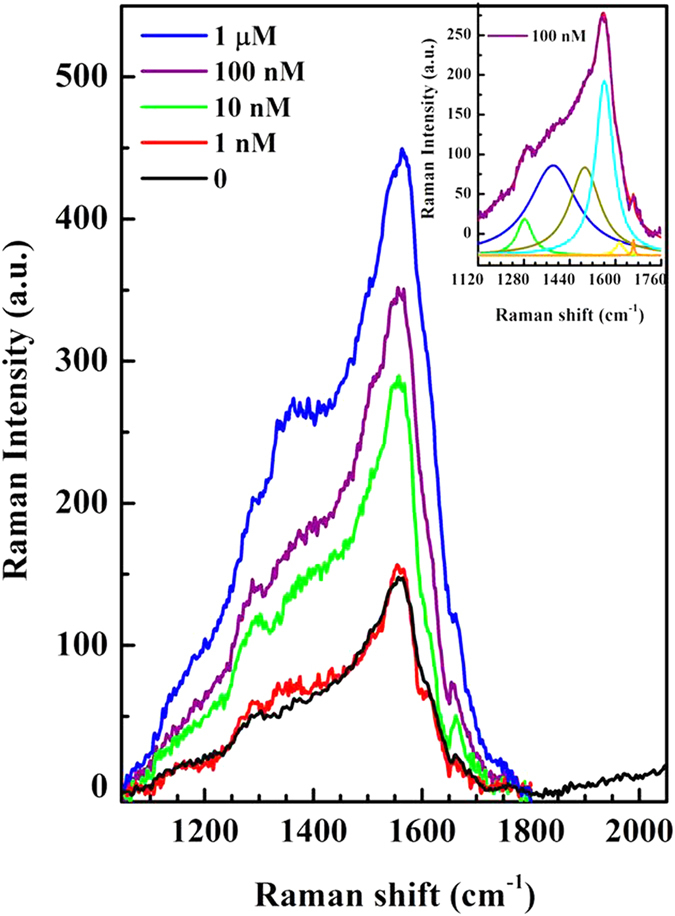
Raman analysis of the biosensor sensitivity. Spectra collected on identical devices at different avidin concentrations, from 1 μM down to 1 nM. The spectrum before protein adsorption is shown as reference (black line). In the inset the result of the fitting procedure applied to the spectrum collected at 100 nM of avidin concentration. The band ascribed to protein adsorption is centered at about 1380 cm^−1^ (blue line).

On the basis of the control test, we explored the sensing ability of the biotin modified Au/SiNW to detect avidin in a wide range of concentration by exploiting the Raman signal without using blocking molecules. Despite the non-perfect uniformity of target molecule adsorption over the whole sensor surface, a definite trend of Raman signal enhancement with protein concentration can be identified. This behavior can be clearly recognized in Fig. 5 showing Raman spectra at different avidin concentrations extending from 1 μM down to 1 nM, as reference we have also plotted the spectrum before protein adsorption (black line). When avidin is added, the intensity of the band at 1380 cm^−1^ increases with the specific concentration of the target solution. The band intensity evolution can be easily quantified by applying a fitting procedure to the collected spectra and used as transduction signal for the biosensor. The figure in the inset exhibits, as an example, the result of the fitting procedure applied to the spectrum collected at 100 nM, demonstrating the presence of the quite large band ascribed to avidin binding (blue line).

### Avidin detection by combined Raman and electrochemical probes

**Figure 6:**
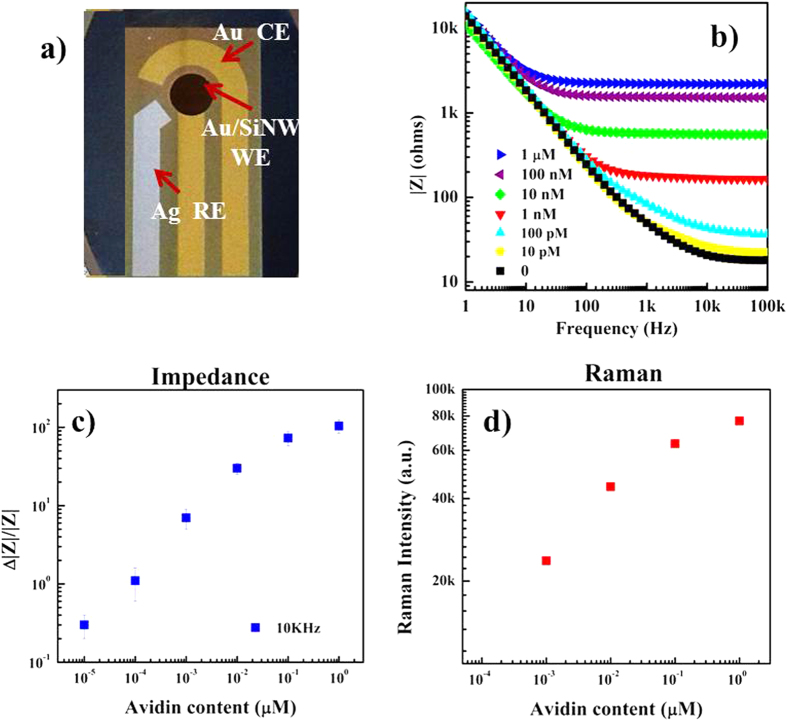
Photograph of the three-electrode device with Au/SiNW used WE and Raman probe (**a**). Bode plots of the impedance modulus, |Z|, for the biotin modified Au/SiNWs after the immersion in avidin solution with different concentration ranging from 10 pM up to 1 μM (**b**). Dependence of impedance variation, 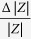, on the avidin concentration obtained at the frequency of 10 KHz. The values correspond to the average measurements performed on a minimum of three samples, identically treated. The relative standard deviation is indicated by the error bars (**c**). Intensity of the Raman band at about 1380 cm^−1^ ascribed to the binding of avidin on the biosensor surface, tuned as for the impedance measurements. The reported values represent the band area obtained by means of a fitting procedure applied to the collected spectra (**d**).

We tested the biosensing capability of the Au/SiNWs to detect avidin through two different and independent transduction signals: Raman and electrochemical via impedance measurements. For this purpose, we exploited the Au coverage wrapping the SiNWs as working electrode (WE) of a three-electrode device, like as shown in Fig. 6a and discussed in more details in ref. 29. This device is simply obtained by adding to the Au/SiNWs, the Au counter electrode (CE) and the Ag reference electrode (RE). We note that the Au/SiNW WE was fabricated in the very same conditions of the above discussed Raman substrates. This configuration enables the collection of both the Raman and electrochemical signal coming from nanostructured WE.

The impedance measurements were performed, after Raman detection, in KCl solution by connecting the electrodes to the potentiostat. The avidin content range was varied between 10 pM and 1 μM, and Fig. 6b shows the related impedance modulus, |Z| measured in the frequency range extending from 100 KHz down to 1 KHz. When avidin is added, a significant and continuous increase of the |Z| values in the whole frequency range analyzed is observed. In Fig. 6c and d we plot, respectively, the impedance response, calculated as 
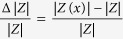
 where |Z(x)| and |Z| are the impedance modulus for x and 0 avidin concentration at the frequency of 10 KHz, where the highest 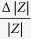 values are observed, and the intensities (area) of the Raman band at about 1380 cm^−1^ ascribed to the binding of the target molecule on the biosensor surface, as function of the avidin content. We observe a very similar trend for both the calibration curves: a liner relationship in the log-log plot in the avidin concentration range of 1 nM-1 μM for Raman transduction and 10 pM-1 μM for impedance signal with saturation at values higher than 1 μM for both the signals.

Concerning the sensitivity limits achieved through the two detection methods, the impedance analysis is characterized by a value as low as 10 pM whereas the Raman signal shows, at the chosen laser power of 3 mW, a higher value of 1 nM. This is due to the fact that for lower concentrations the Raman signal would require a higher excitation power, associated to a different calibration curve, but such as not to damage and alter the biological sample. Moreover, we expect an improvement of performance for both the sensing responses by optimizing some key material features, including the NW size, Au coverage thickness and uniformity. Nevertheless the low detection limit and working range found are among the highest reported in the literature. This comes from the comparison of our results with those in Table 1, where some works reported for the (strept)avidin detection based on nanosized materials via optical or electrochemical approach are summarized. In addition our biosensor benefits the unique advantage to couple the SERS chemical information with the electrochemical quantification that increases the analysis efficiency and enlarges the application areas over the common approach based on detection via one transduction mechanism.

## Conclusion

We explored forest of SiNWs covered by a Au layer as effective substrate for enhancing Raman signal of the adsorbed molecules. We showed several unique features of these systems. First, the NW morphology imparts an enhancement of light absorption in the overlaying Au with the occurrence of plasmonic peaks resonant to the wavelength of Raman excitation source. Second, the disordered arrangement of the NWs offers the advantage to use high yield techniques, such as PECVD, that involve relative low temperature procedures compatible with non-conventional substrate such as microscope glass slides, rapidly implementable in Raman spectrophotometers. Third, the design involving a conductive coverage of the SiNWs allows developing innovative devices able to detect one analyte through both Raman and impedance transduction signals by increasing the analysis efficiency and reducing the detection issues related to nonspecific interaction. In conclusion, the proposed composite system not only is efficient Raman substrate, fully compatible with the industrial integrated silicon based technology, but also can be designed aiming at multi transduction methods, thus putting in perspective the application of SERS in innovative analytical contexts.

## Methods

### Fabrication of SiNWs

Au catalyzed SiNWs were produced by PECVD on both microscope glass and Si wafer covered by 1 μM of thermal SiO_2_. To induce the NW growth, a 2 nm thick Au film was selectively evaporated onto the specific area, defined using conventional photolithography and wet etching processes. The growth was performed with SiH_4_ and H_2_ as precursor at a total pressure of 1 Torr and substrate temperature of 350 °C. The flow ratio SiH_4_/(H_2_ + SiH_4_) was fixed to 1:10. A 13.6 MHz radiofrequency with power fixed at 5 W was used to ignite the plasma. The growth time was 7 min. The Au coverage, 150 nm thick, was evaporated onto the specific areas sequentially defined by UV-lithography. The three-electrode device was fabricated on Si/SiO_2_ as reported in ref. 29.

### Surface functionalization of SiNWs

The bio-sensing tests were performed using the biotin-avidin scheme. The immobilization of the NHS-biotin group onto the Au/SiNWs was performed *via* the modification of the Au/SiNWs surface with SAM by immersing the samples in cysteamine solution 20 mM and incubating them overnight at 70 °C. They were rinsed off the residual cysteamine molecules with deionized water, Raman (and also impedance in the case of the three-electrode device) spectra were measured to ensure that the cysteamine molecules had been linked to the NW surface. The cysteamine functionalized samples were immersed in a fresh solution of 10 mM NHS-biotin in pH 7.4 phosphate-buffered saline (PBS) solution for 60 min at room temperature, and then cleaned with PBS. Also in this case, the immobilization of NHS-biotin onto cysteamine was controlled by Raman (impedance) investigation. Afterwards, the biotin-functionalized Au/SiNWs were immersed in 1.5 mL of aqueous avidin solution with different concentrations ranging from 1 μM down to 10 pM for 30 min at room temperature, rinsed again in PBS and dried before being tested. For each avidin concentration fresh samples were used and the experiments were sequentially conducted after cysteamine coverage, NHS-biotin modification and avidin detection. In order to treat with the biomolecules only the area having the NWs, we delimited it with removable Teflon wells fixed on the NW area under mild pressure.

### Morphological and optical characterization

The morphology of the SiNWs was verified by field emission scanning electron microscopy (FE-SEM), equipped by an energy dispersive X-ray spectrometer for composition analysis. The SEM images were acquired at an accelerating voltage of 5 kV. The EDX spectra were measured at 10 keV. The optical properties were studied by measuring the angle-integrated total reflectivity in the spectral range between 200 and 2500 nm with UV-Vis-IR spectrophotometer equipped with an integrating sphere.

### Raman spectroscopy

Raman spectra were acquired with a DXR Thermo Fisher Scientific Raman Microscope, by exciting the samples at 532 nm with a 5 mW power and a 50× objective. The measured spectral range was 50–3000 cm^−1^, and each spectrum resulted from 1 s acquisition time and 200 accumulations over an area of about 800 nm (laser spot). The spectra were analysed performing a lorentzian fit to obtain the values of the band intensities calculated as the area of the selected spectral features.

### Impedance measurements

The impedance measurements were performed on the three-electrode device at room temperature in a Faraday cage at DC 0 V and with an AC signal of 10 mV in a stimulus range between 30 mHz–100 KHz. The tests were carried out in a solution 100 mM of KCl in deionized and distilled water. A test circular area of 1.5 cm^2^, with the Au/SiNW WE at the center of that area and part of RE and CE immersed in the solution, was delimited by using a Teflon tube. In order to avoid solution leakage, an O-ring was placed between the test sample and the bottom of the Teflon tube. The potentiostat used was a versaSTAT 4 by PAR. Each measurement was performed with a minimum of four repetitions on the same electrode over a time period of 1 h.

## Additional Information

**How to cite this article**: Convertino, A. *et al.* Disordered array of Au covered Silicon nanowires for SERS biosensing combined with electrochemical detection. *Sci. Rep.* **6**, 25099; doi: 10.1038/srep25099 (2016).

## Figures and Tables

**Table 1:** Comparison of various biosensors based on nanosized materials detecting (strept)avidin via optical or electrochemical methods.

Ref.	Sensing Material	Signal Transduction	Target	Low detection limit	Working range
Cui 2001^24^	SiNW FET*	Conductance	Streptavidin	10 pM	
Haes 2002^37^	AgNPs	SPR§	Streptavidin	1 pM	1 pM–100 pM
Cheng 2003^38^	AuNPs/optical fiber	SPR	Streptavidin	100 pM	100 pM–30 nM
Kim. 2007^39^	μAg particles	SERS	Avidin	15 nM	15 nM–10 μM
Hsiao 2009^40^	Poly–SiNW FET	Conductance	Streptavidin	0.17 pM	0.17 pM–1.7 nM
Hong, 2010^41^	Ordered Macroporous Ag thin films	SPR	Avidin	100 pM	100 pM–200 nM
Choi 2010^42^	ZnO NW FET	Conductance	Streptavidin	2.5 nM	2.5 nM–25 nM
Guo 2010^43^	Au NP/CNT	SPR	Streptavidin	500 pM (calculated)	1 nM–400 nM
Geagea 2015^44^	Polymer coated AgNPs	Impedance	Avidin	1.5 nM	

^*^field effect transistor (FET).

^§^surface plasmon resonance (SPR).
